# Reversal of MicroRNA Dysregulation in an Animal Model of Pulmonary Hypertension

**DOI:** 10.1371/journal.pone.0147827

**Published:** 2016-01-27

**Authors:** Igor B. Gubrij, Amanda K. Pangle, Li Pang, Larry G. Johnson

**Affiliations:** 1 Central Arkansas Veterans Healthcare System, Little Rock, Arkansas, United States of America; 2 Department of Internal Medicine, Division of Pulmonary and Critical Medicine, The University of Arkansas for Medical Sciences, Little Rock, Arkansas, United States of America; 3 Department of Pharmacology, The University of Arkansas for Medical Sciences, Little Rock, Arkansas, United States of America; Vanderbilt University Medical Center, UNITED STATES

## Abstract

**Background:**

Animals models have played an important role in enhancing our understanding of the pathogenesis of pulmonary arterial hypertension (PAH). Dysregulation of the profile of microRNAs (miRNAs) has been demonstrated in human tissues from PAH patients and in animal models. In this study, we measured miRNA levels in the monocrotaline (MCT) rat model of PAH and examined whether blocking a specific dysregulated miRNA not previously reported in this model, attenuated PAH. We also evaluated changes in miRNA expression in lung specimens from MCT PAH rats overexpressing human prostacyclin synthase, which has been shown to attenuate MCT PAH.

**Methods:**

Expression levels of a panel of miRNAs were measured in MCT-PAH rats as compared to naïve (saline) control rats. Subsequently, MCT PAH rats were injected with a specific inhibitor (antagomiR) for miR-223 (A223) or a nonspecific control oligonucleotide (A-control) 4 days after MCT administration, then weekly. Three weeks later, RV systolic pressure and RV mass were measured. Total RNA, isolated from the lungs, microdissected pulmonary arteries, and right ventricle, was reverse transcribed and real-time quantitative PCR was performed. MiRNA levels were also measured in RNA isolated from paraffin sections of MCT-PAH rats overexpressing prostacyclin synthase.

**Results:**

MiRs 17, 21, and 223 were consistently upregulated, whereas miRs 126, 145, 150, 204, 424, and 503 were downregulated in MCT PAH as compared to vehicle control. A223 significantly reduced levels of miR-223 in PA and lungs of MCT PAH rats as compared to levels measured in A-control or control MCT PAH rats, but A223 did not attenuate MCT PAH. Right ventricular mass and right ventricular systolic pressure in rats treated with A223 were not different from values in A-control or MCT PAH rats. In contrast, analysis of total RNA from lung specimens of MCT PAH rats overexpressing human prostacyclin synthase (hPGIS) demonstrated reversal of MCT-induced upregulation of miRs 17, 21, and 223 and an increase in levels of miR-424 and miR-503. Reduction in bone morphogenetic receptor 2 (BMPR2) messenger (m)RNA expression was not altered by A223, whereas human prostacyclin synthase overexpression restored BMPR2 mRNA to levels in MCT PAH to levels measured in naive controls.

**Conclusions:**

Inhibition of miR-223 did not attenuate MCT PAH, whereas human prostacyclin synthase overexpression restored miRNA levels in MCT PAH to levels detected in naïve rats. These data may establish a paradigm linking attenuation of PAH to restoration of BMPR2 signaling.

## Introduction

Pulmonary arterial hypertension (PAH) is a devastating disease leading to progressive right-sided heart failure and potentially death. Approximately 10–15% of patients with PAH die within one year of medical follow-up despite treatment [1–3]. Thus finding new therapies to treat this disorder remains a challenge for contemporary medical science.

Epigenetic regulation of PAH pathogenesis with small non-coding RNAs of about 20–25 nucleotides, known as microRNAs (miRNA), has been gaining increasing attention. These non-coding RNAs bind imperfectly to the 3’ UTR of target mRNAs to regulate the expression of target genes post-transcriptionally. Dysregulation of more than a dozen miRNAs has been reported in PAH lung specimens from humans and animal models [4–10]. The expression of specific miRNAs involved in angiogenesis and vascular remodeling in plexiform arteriopathy has been investigated in lung specimens from patients with severe PAH [11]. Plexiform lesions (PLs) are complex vascular lesions that may arise from (monoclonal) proliferation of ECs, migration and proliferation of SMCs and accumulation of circulating cells, e.g., macrophages and endothelial progenitor cells. They are generally associated with concentric obliterative lesions (CLs) characterized by concentric intimal fibrosis and prominent hypertrophy of the vascular media. Differential expression of miRs has been reported in lung specimens of patients with severe IPAH or APAH undergoing lung transplantation with samples from the lung allograft downsized for transplantation used as controls [11]. Levels of miR-143/145 expression were increased in CLs from PAH subjects as compared to control specimens from control unremodeled PA, whereas MiR-143/145 expression was decreased in PLs as compared to CLs and unremodeled control PA. MiR-126 expression was decreased in PLs and CLs from PAH subjects relative to unremodeled control PA, but miR-126 expression was nevertheless significantly higher in PLs than in CLs of PAH specimens. Downregulation of miR-204 also occurred in PLs and CLs with upregulation of miR-21 in both PLs and CLs. The data were generally consistent with published data from others in human PAH specimens [12–15].

Additional data from human specimens and in hypoxia and monocrotaline-induced PAH specimens further establish that all three layers of the pulmonary artery—endothelium, muscle, and adventitia—are affected by dysregulation of miRNA expression in PAH. Among these the miR-17–92 cluster, miR-21, miR-145, miR-204 and miR-210 are dysregulated in PASMCs, miR-124 is primarily dysregulated in fibroblasts in the adventitia, and miR-17, miR-21, miR-424, and miR-503 appear to play important roles in PAECs. Importantly, miR-21 and the miR-17-92 cluster have been shown to be involved in BMPR2 down-regulation. The miR-17–92 cluster also regulates several proteins involved in cell cycle progression, such as E2F1 and p21 [10].

Several studies have shown that inhibition or enhancement of dysregulated miRNA expression can attenuate experimental pulmonary hypertension. For example, inhibition of miR-17 attenuated PAH in both the MCT and chronic hypoxia PAH models. In contrast, miR-21 expression may vary between chronic hypoxia, monocrotaline, and hypoxia/SU5416 models of PAH with downregulation such that either enhancement of miR-21 expression with miR mimetics (Hypoxia/SU5416 model) or downregulation of miR-21 expression with inhibitors (MCT model and some hypoxia models) may be beneficial [16–18]. Inhibition of miR-145 [12], enhancement of miR-328 expression [14], and enhancement of the miR424/503-FGF axis also attenuated experimental pulmonary hypertension [19, 20].

Multiple signaling pathways have been associated with PAH pathogenesis including TGFβ/BMPR2, RhoA/RhoB kinase, serotonin, Notch, NO/cGMP, endothelin, Angiopoietin/Tie2, and the APLN/FGF2/FGFR1 signaling pathways [4, 21–23]. MiR-17, miR-21, and miR-145 dysregulation have been linked to TGFβ, BMPR2 and RhoA/RhoB kinase signaling [24–26]. MiRs 204 and 206 have been linked to Notch, PDGF, endothelin, Ang/Tie2, and Stat-3 signaling [25, 26], and miRs 424 and 503 have been linked to APLN/FGF_2_/FGFR1 and Stat-3 signaling [20].

In this study, we examined the expression of a panel of miRNAs in the monocrotaline (MCT) PAH rat model, evaluated the functional role of a specific miR-223 inhibitor on attenuation of PAH, and determined the results of human prostacyclin synthase (hPGIS)-mediated attenuation of MCT PAH on this miRNA panel. The panel of miRNAs selected for study were based on experimental evidence for involvement of the miRNA in both human and experimental models of PAH. MiR-223 has previously been considered a myeloid specific miRNA, but has been shown to be dysregulated in cardiovascular medicine with a potential role as a biomarker of acute myocardial infarction and heart failure [9,10]. It is also upregulated in lungs of smokers with COPD [27]. Because its role in PAH is unknown, we selected it as the miRNA for selective downregulation in our animal model. Finally, we evaluated the effects of these interventions on BMPR2 mRNA levels and also on IGF1R mRNA levels, which TargetScan predictions identify as a target for miR-223.

## Materials and Methods

### AntagomiR synthesis

MiRNA-223 inhibitor (antagomiR-223, designated below as A223) was synthesized and purchased from Exiqon, Inc., as an LNA^™^-enhanced oligonucleotide that contained phosphorothioate backbone for optimal use in miRNA functional studies. Its sequence (5’-ATTTGACAAACTGAC-3’) is complimentary to miR-223-3p. A nonspecific control oligonucleotide, 5’-ACGTCTATACGCCCA-3’, was used as a negative control (A-control).

### Animals and experimental design

All animal experiments and procedures were approved by the Institutional Animal Care and Use Committees of the Central Arkansas Veterans Healthcare System. Female Sprague Dawley rats (age 6–8 weeks) were purchased from Charles River Laboratories. Seven days after arrival, animals were intraperitoneally injected with MCT (60 mg/kg) or an equal volume of saline. The tail veins of MCT treated rats were injected with A223, A-control, or saline on days 4 and 9 (15 mg/kg), and 14 (7.5 mg/kg) after MCT injection. Right ventricular systolic pressure (RVSP) and right ventricle/left ventricle + septum (RV/LV+S) ratios were measured 21–24 days after MCT injection. The dose of antagomiR delivered in this study closely falls in the mid range of 2–25 mg/kg delivered parenterally at least weekly by Caruso, Pullamsetti, and others [8, 12, 15].

### Measurement of right ventricle mass and right ventricle pressures

The right internal jugular vein of anesthetized rats was surgically exposed and cannulated with a 2F pressure transducer catheter (Millar Instruments) interfaced to a control unit (PC-2000) and a data acquisition system (Power Lab 8/30, AD Instruments). The catheter was advanced into the right ventricle (RV), and the RVSP was recorded. After euthanasia, the hearts of all experimental animals were removed, blotted, and the RV was dissected free of the left ventricle plus septum (LV+S). Each portion was then weighed.

### Measurement of miRNA expression by quantitative PCR

Tissues from the lung, RV and microdissected pulmonary artery (PA) were collected immediately after euthanasia, placed into RNAlater Solution (Ambion/Life Technologies), and stored at -20°C. Collected blood plasma was frozen right away. Total RNA purification was performed after tissue homogenization using the mirVana miRNA Isolation Kit (Ambion/Life Technologies) following manufacturer recommendations. Plasma RNA was isolated from 0.2 ml plasma employing similar procedure. We also isolated total RNA from formalin fixed paraffin embedded lung sections from a prior study that demonstrated functional attenuation of MCT PAH by overexpression of (hPGIS) in rat lungs for measurement of miRNA expression [28]. Total RNA from the paraffin embedded lung sections was isolated by means of the Recover All^™^ Nucleic Acid Isolation kit (Ambion/Life Technologies) as recommended by the manufacturer. MiRNA expression levels were evaluated in two-step procedure. First, reverse transcription was performed with specific primer sets for each miRNA, which were provided with the TaqMan MicroRNA Assays, utilizing the TaqMan MicroRNA Reverse Transcription Kit (Applied Biosystems/Life Technologies). Total RNA (10 ng) was added to each reverse transcription reaction. Real-Time quantitative TaqMan polymerase chain reaction (qPCR) was performed with TaqMan MicroRNA Assays and TaqMan Universal Master Mix II (Applied Biosystems/Life Technologies), adding 4.3 μl reverse transcription product per 20 μl reaction. PCR cycles were conducted on an Applied Biosystems 7900HT Fast Real-Time PCR System. The levels of miRNA expression were calculated by ΔΔCt method. Expression levels of miR-17-5p, miR-21-5p, miR-126-3p, miR-145-5p, miR-150-5p, miR-204-5p, miR-223-3p, miR-328-3p, miR-424-5p (mmu-miR-322, the mouse/rat ortholog for hsa-miR-424), and miR-503-5p were evaluated. As internal controls, U6 small nuclear RNA for tissue RNAs, and miR-103 for plasma RNAs were used. A portion of our plasma samples were supplemented with 5 nmol/L Caenorhabditis elegans miR-39 (cel-miR-39) for the normalization of RNA preparations to validate our results as compared to the miR-103 internal control [29, 30].

### Quantification of BMPR2 and IGF1R mRNA expression

Evaluation of BMPR2 messenger (m)RNA expression in the lungs and PA of experimental animals was performed using total RNA preparations. For this analysis, TaqMan Gene Expression Assays for BMPR2 and IGF1R (with β-actin as an internal control), and TaqMan RNA-to-CT^™^ 1-Step Kits (Applied Biosystems/Life Technologies) were employed in Real-Time qPCR performed on 7900HT Fast Real-Time PCR System (Applied Biosystems). The results were calculated using ΔΔCt method.

### Statistical analysis

Data are presented as the mean ± SE. The data were analyzed with the application of the One Way ANOVA method. A *p* value < 0.05 was considered statistically significant.

## Results

### MiRNA expression in the MCT rat model of PAH

**Fig 1:**
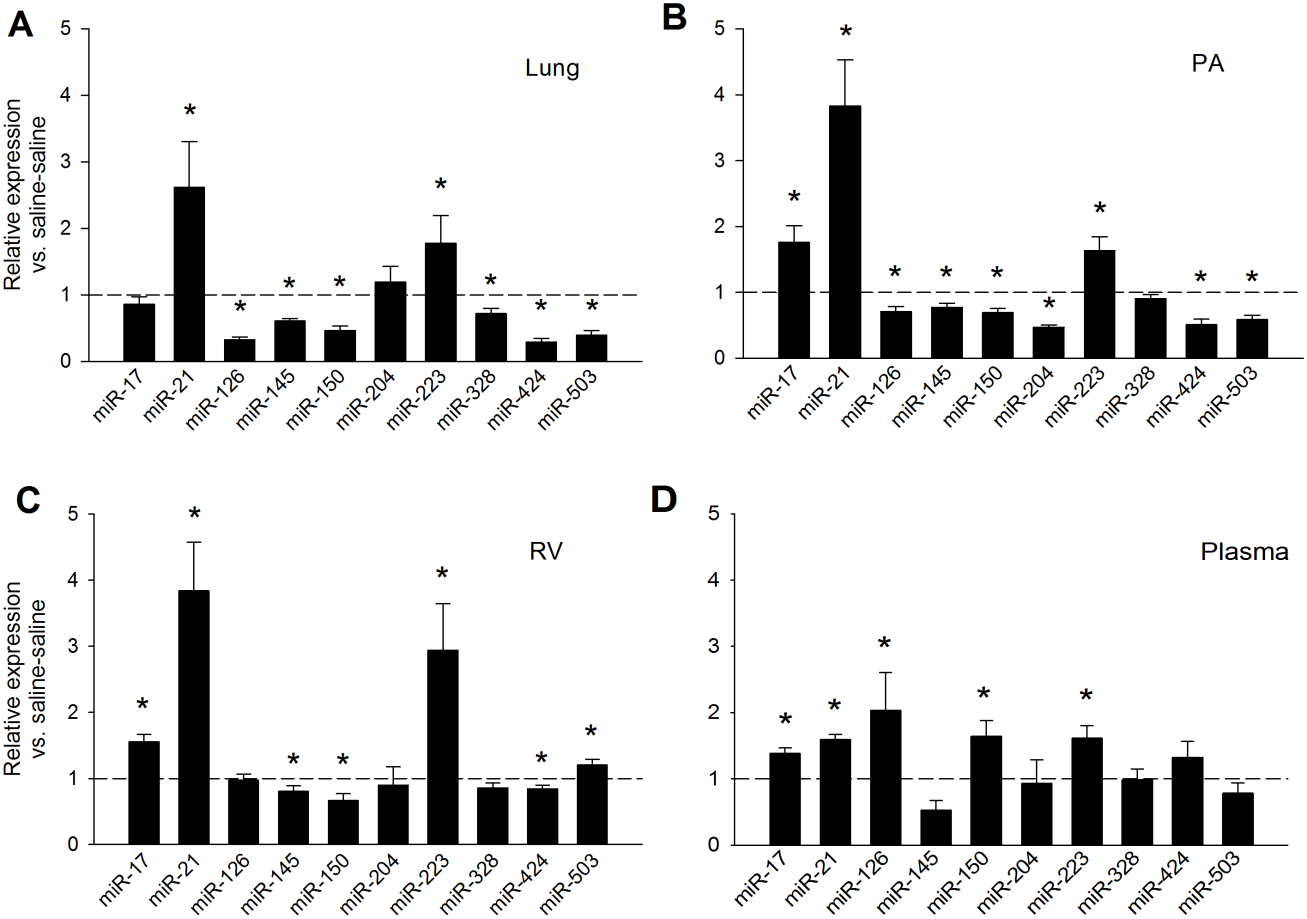
MiRNA expression in MCT PAH rats. Expression levels were measured 21–24 days after MCT treatment and are relative to saline-saline (naïve) control animals. (A) Lungs. (B) PA. (C) RV. (D). Plasma. *Significantly different from saline-saline control; n = 8. --- Saline-saline control reference line.

We examined the changes in expression levels of ten miRNAs using MCT PAH rats. The measurements were performed in lung, PA, RV, and plasma. MCT PAH rats displayed a significant increase of miR-21 and miR-223 in all tissues evaluated (Fig 1). A group of miRNAs were downregulated in the lung and PA of MCT PAH rats, including miR-126, miR-145, miR-150, miR-424, and miR-503 (Fig 1A and 1B). Levels of miR-145 were also reduced in the RV (Fig 1C). MiR-17 was not changed in the lung, but increased in the RV and PA (Fig 1B and 1C). Noteworthy was a >3 fold increase in miR-126 in plasma from MCT PAH rats compared to vehicle control rats (Fig 1D). This increase was accompanied by significant increases in miR-17, 21 and 223 expression levels in plasma relative to vehicle controls (Fig 1D), whereas miR-145 was downregulated in plasma (Fig 1D). MiR-150 was downregulated in the RV and, surprisingly, upregulated in plasma (Fig 1C and 1D). MiR-204 was decreased only in the PA of MCT PAH rats compared to controls, and miR-328 was significantly reduced only in the lungs of MCT PAH rats (Fig 1A). MiR-424 (miR-322 ortholog) was decreased ~2 fold in the lungs and PA (Fig 1A and 1B) and decreased only slightly in RV, while miR-503 slightly increased in RV with no change in plasma (Fig 1C and 1D). The upregulation of miR-21 and miR-223 for all tissues examined indicates the possibility they are ubiquitously upregulated in all tissues affected by PAH, while other miRNAs may have a more tissue-specific dysregulation, for example miR-124 which is predominantly dysregulated in adventitial fibroblasts.

**Fig 2:**
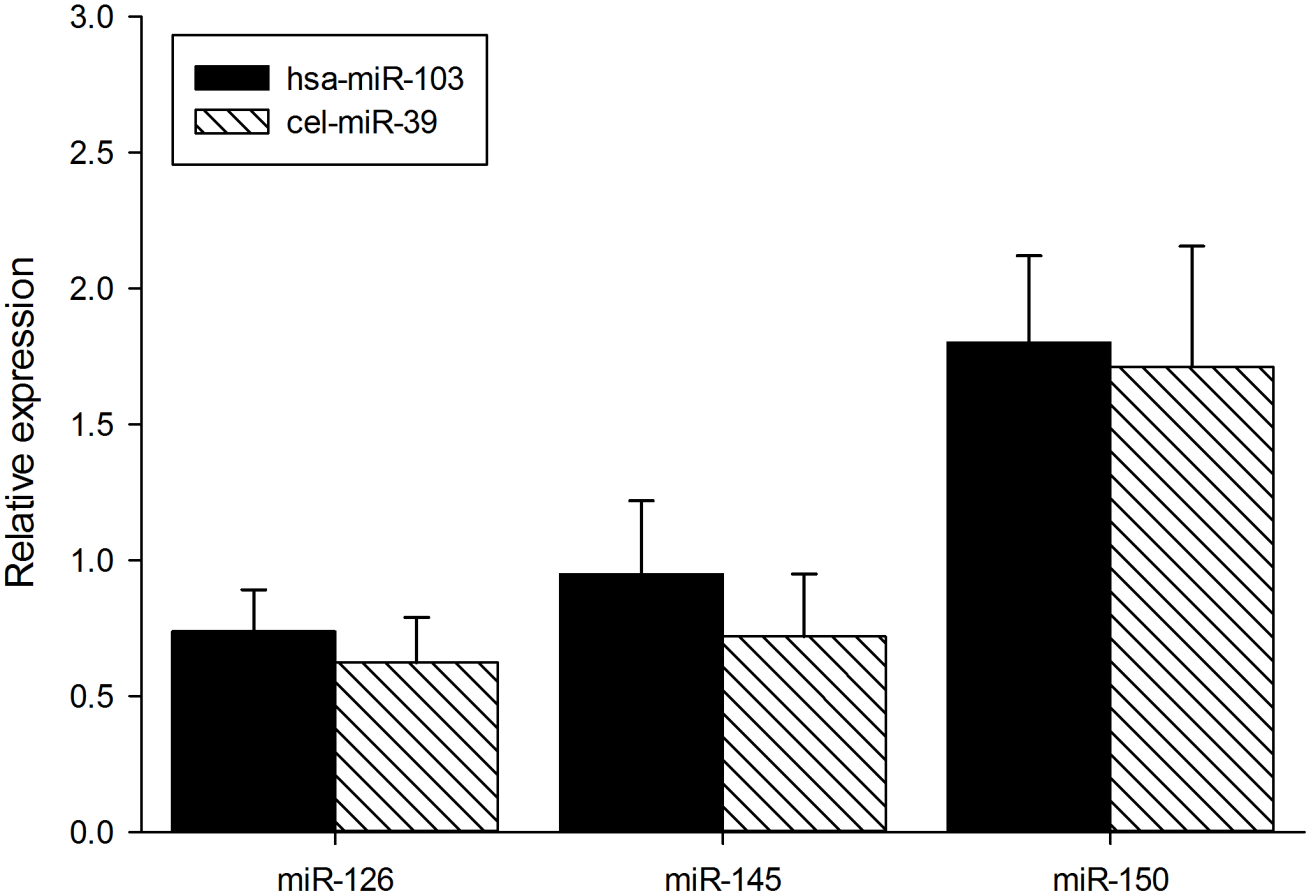
Comparison of normalization methods in plasma. MiR-103 and c. elegans miR-39 normalization methods were compared in plasma from MCT PAH rats. N = 4. p values are as follows: MiR-126 p = 0.499, miR-145 p = 0.422, miR-150 p = 0.803.

To validate measurements of circulating miRNAs in plasma normalized with an internal miR-103 standard, we supplemented fresh plasma from MCT PAH and naïve (saline control) rats with a C. elegans miR-39 standard and measured levels of miR-126, miR-145 and miR-150 in plasma from MCT PAH rats and saline control rats. As shown in Fig 2, the levels of miR-126, 145 and 150 were similar when normalized to either a C. elegans miR39 standard or an internal miR-103 control (Fig 2).

### Specific inhibition of miR-223 in MCT PAH rats

**Fig 3:**
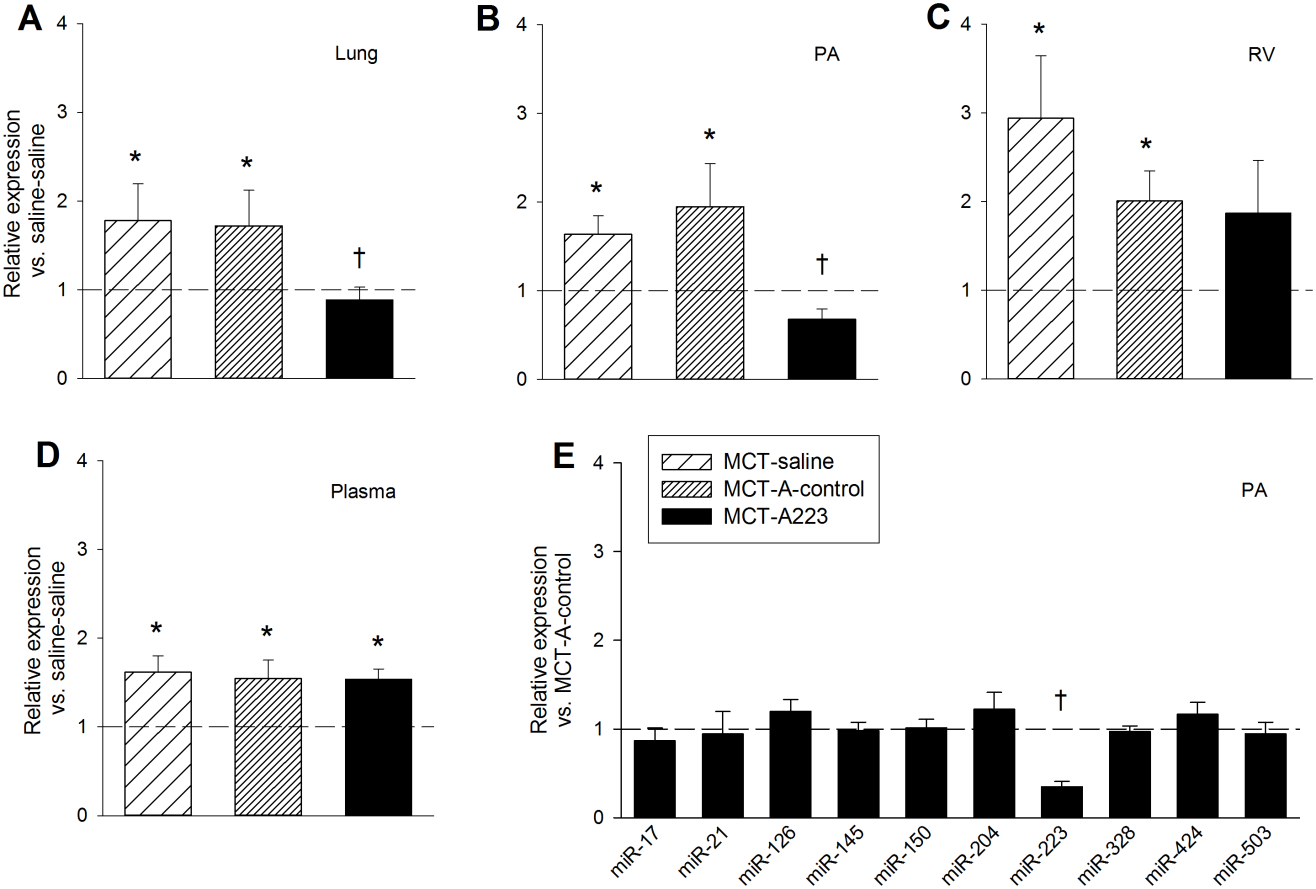
Effect of A223 administration on miRNA expression in MCT PAH. Expression levels of miR-223 relative to saline-saline (naïve) control animals. (A) Lungs. (B) PA. (C) RV. (D) Plasma.—Saline-saline control reference line. (E) Expression of miRNAs in the PA of MCT-A223 animals relative to MCT-A-control animals.—MCT-A- control reference line.*Significantly different from saline-saline control; ^**†**^Significantly different from MCT-saline and MCT-A- control; n = 8.

**Fig 4:**
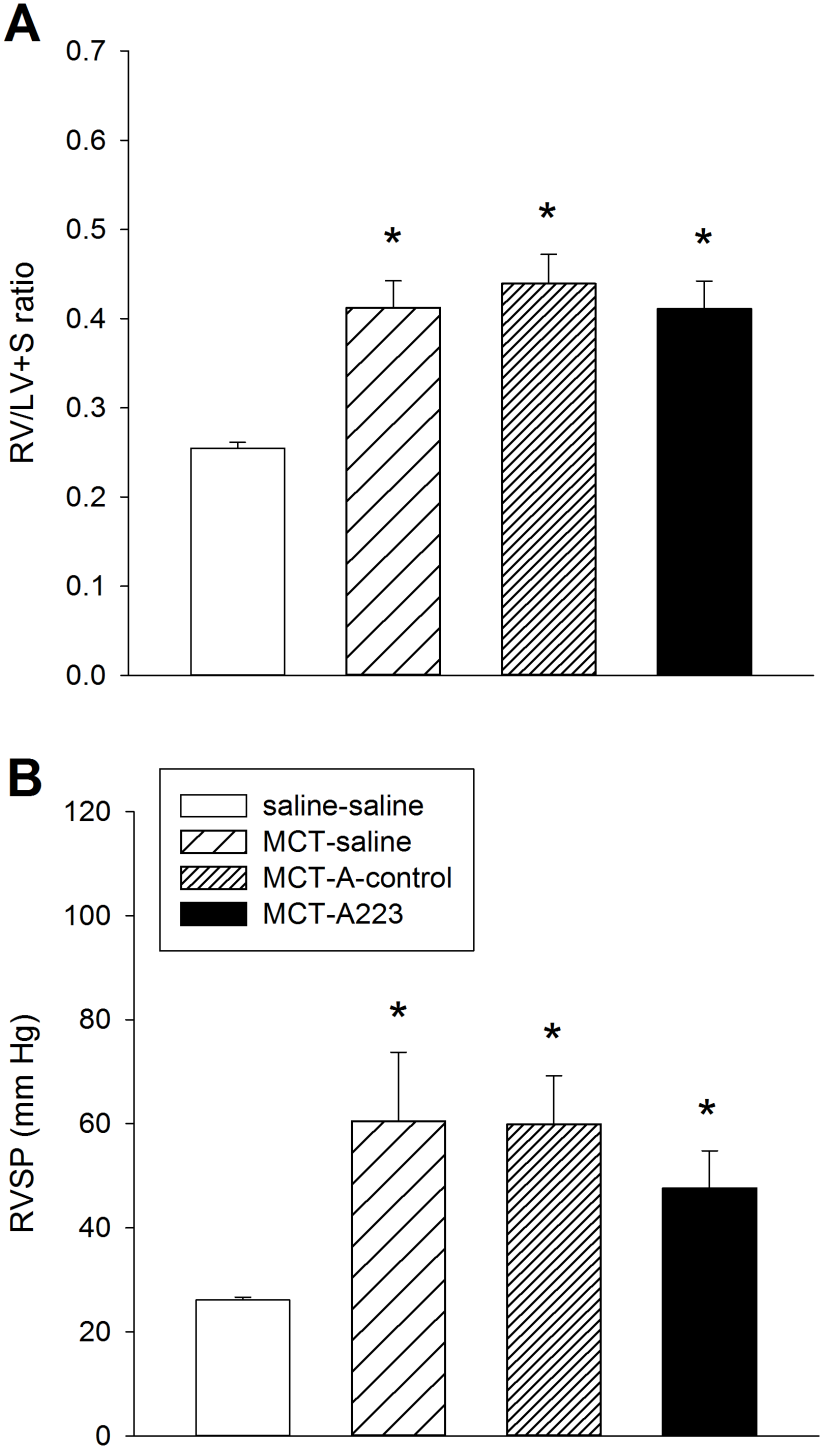
Functional assessment of A223 treatment in rats after MCT administration. (A) RV/(LV+S) ratios, n = 8. (B) RVSP measurements, n = 4–7. *Significantly different from saline-saline control.

To explore whether findings from miR-223 dysregulation in cardiovascular disease could be extended to PAH [31, 32], we measured expression of miR-223 in MCT PAH rats. As shown in Fig 1, miR-223 was upregulated in lung, PA, RV, and plasma of MCT PAH rats. To evaluate a potential pathogenic relationship between miR-223 upregulation and PAH, we performed specific inhibition of this miRNA by injecting MCT PAH rats with A223 or a nonspecific control oligonucleotide, A-control. A223 significantly decreased miR-223 expression in the lung and PA, but not RV, of MCT PAH rats to levels measured in vehicle controls (Fig 3A–3D), whereas A-control did not. The A223-mediated reduction in levels of miR-223 in MCT PAH rats was specific, as levels of the remaining miRNAs in our panel were unchanged (Fig 3E). The functional effects of specific inhibition of miR-223 by A223 on MCT PAH were also evaluated. Measurements of RV/LV+S ratios and RVSP were not different in A223 treated MCT PAH rats from values measured in MCT PAH rats treated with A-control or vehicle (Fig 4), consistent with failure to attenuate MCT induced PAH.

### MiRNA profile changes induced by AAV-mediated expression of hPGIS in MCT PAH

**Fig 5:**
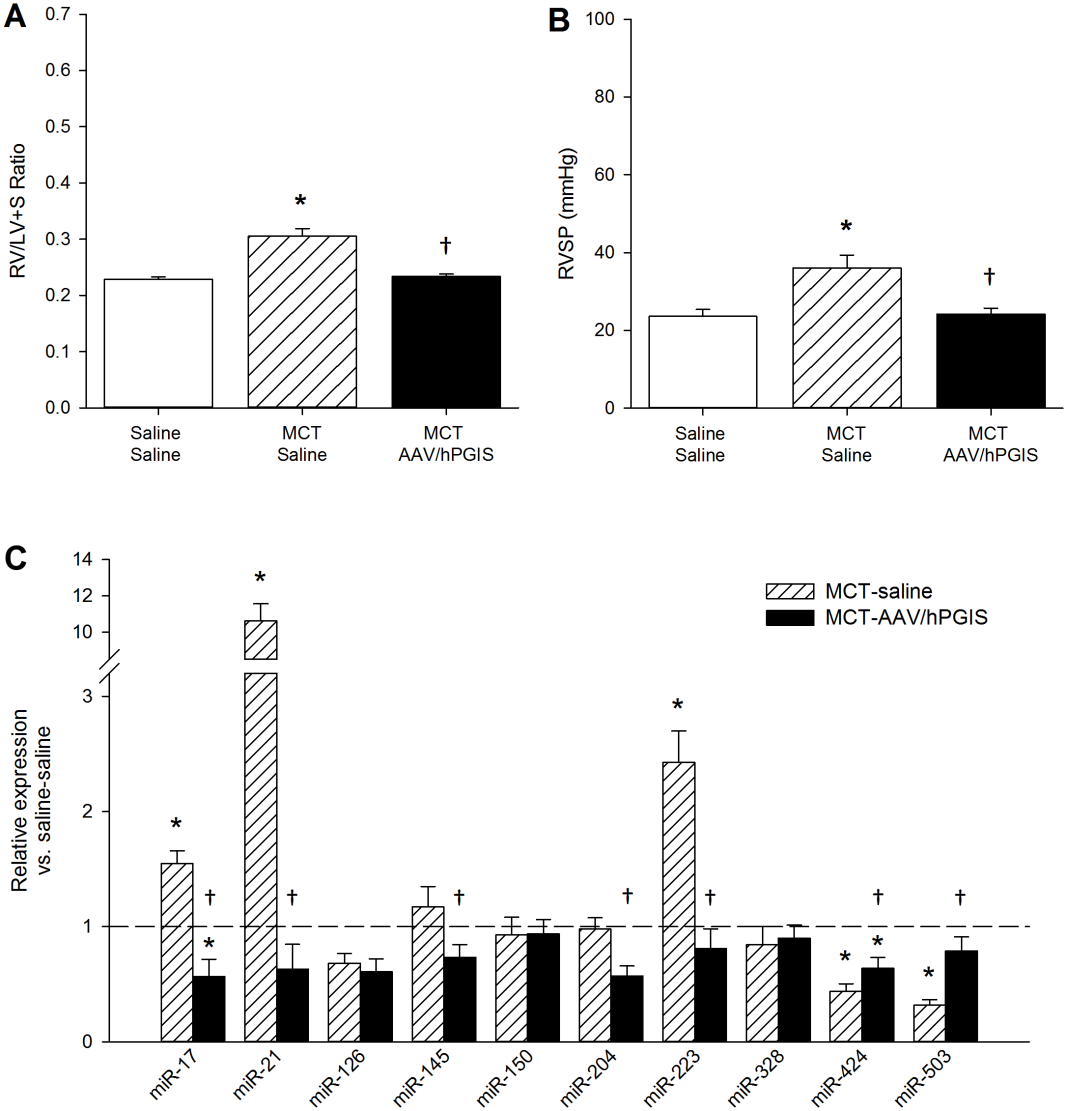
Assessment of AAV-mediated overexpression of hPGIS on miRNA expression in the formalin-fixed paraffin-embedded lung specimens from MCT PAH. Functional assessment of AAV-mediated overexpression of hPGIS in MCT PAH rats. (A) RV/(LV+S) ratios. (B) RVSP measurements. (C) MiRNA expression in lung specimens from MCT-saline and MCT-AAV-hPGIS rats relative to saline-saline (naïve) control; *Significantly different from saline-saline control; ^**†**^Significantly different from MCT-saline control; n = 4.—Saline-saline control reference line. Hemodynamic data is adapted from reference [28].

Previously we reported functional correction of MCT induced PAH by overexpression of hPGIS with AAV vectors [33]. Overexpression of hPGIS restored RV mass (RV/LV+S ratios) and RVSP in MCT PAH rats to levels detected in naïve rats (Fig 5A and 5B). We isolated total RNA from residual paraffin embedded lung specimens from that study and performed qPCR for our miRNA panel. Lung specimens from MCT control rats exhibited a miRNA expression profile similar to the pattern detected in initial experiments (Figs 1A and 5). In contrast, the miRNA panel from lung specimens of MCT rats overexpressing hPGIS exhibited restoration of dysregulated miRNA levels to levels of naïve control rats, with downregulation of miRNAs that had been increased by MCT (miR-17, miR-21, and miR-223), and upregulation of miRNAs that had been decreased by MCT (miRs 424 and 503), Fig 5C]. Subtle differences in the panel from paraffin sections compared to the panel from fresh specimens were noted. MiR-126, 145, 150, and 328 were not significantly reduced in the paraffin sections as compared to the fresh specimens, whereas miR-17 was significantly increased in the paraffin sections as compared to the fresh specimens. These findings may reflect strain differences since the current study uses Sprague Dawley rats whereas the paraffin embedded specimens were collected from Fischer 344 rats, or alternatively, differences in statistical power given the small number of paraffin sections available for study.

### Role of BMPR2 signaling in attenuation of MCT induced PAH

**Fig 6:**
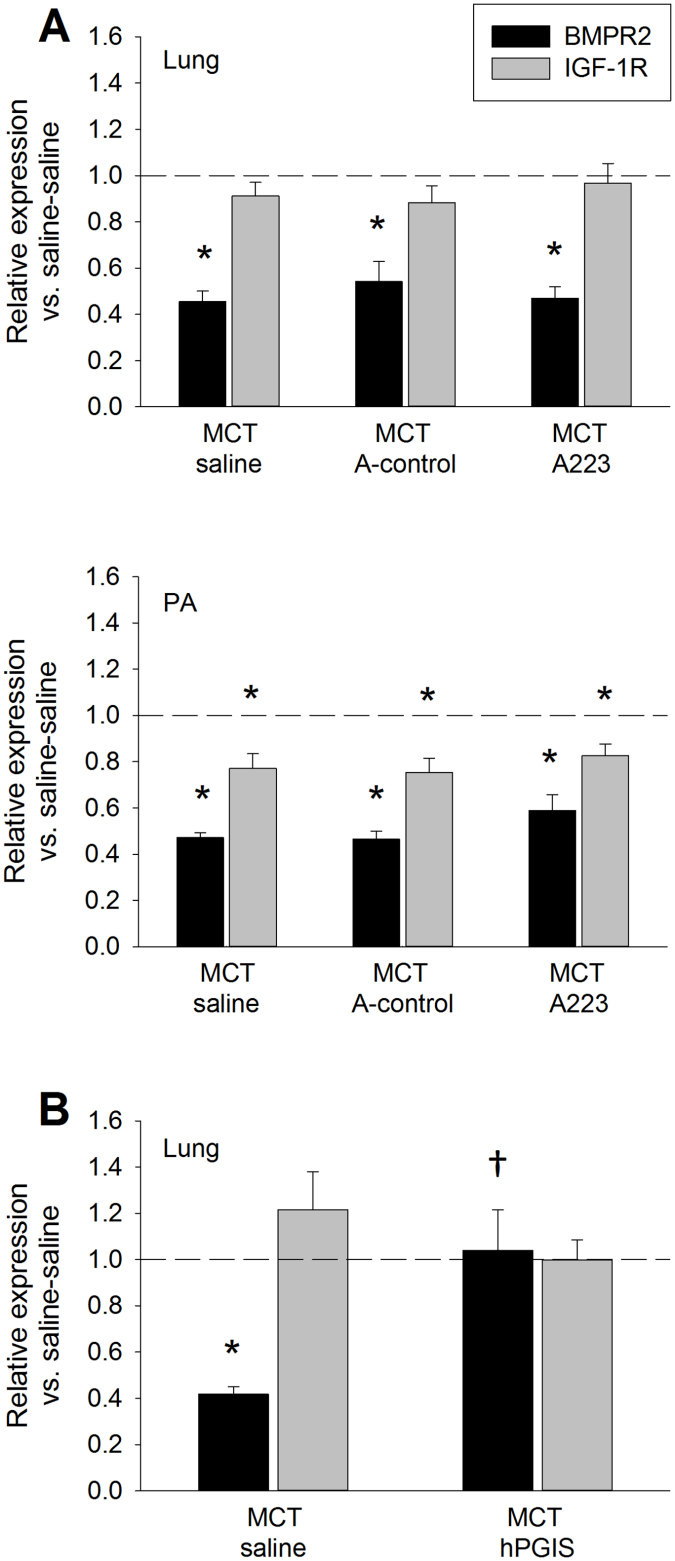
Changes in BMPR2 and IGF-1R mRNA expression in MCT PAH. (A) Expression in lungs and PA after A223 administration, n = 8. (B) Expression in lungs after hPGIS overexpression mediated attenuation of MCT PAH, n = 4. *Significantly different from saline-saline control; ^**†**^Significantly different from MCT-saline control.—Saline-saline control reference line.

Since miRs 17, 21, and 223 were significantly upregulated in the PA of MCT PAH rats, we performed qPCR for BMPR2 and IGF1R mRNA, as these miRNAs have been associated with downregulation of BMPR2 (miR 17, 21 and 145) and IGF1R (miR-223 and 328). Levels of BMPR2 mRNA were significantly reduced in lung and PA of MCT PAH rats, while levels of IGF1R mRNA were reduced in PA, but not lung, of MCT treated rats (Fig 6A and 6B). A223 administration did not alter the reduction in BMPR2 levels induced by MCT, nor did it have an effect on levels of IGF1R. In contrast, BMPR2 mRNA levels in paraffin embedded lung specimens of MCT rats with AAV mediated overexpression of hPGIS were restored to levels measured in lungs of naïve control animals not treated with either MCT or AAV vector (Fig 6C). Overexpression of hPGIS had no effect on IGF1R mRNA levels.

## Discussion

In the current study, we evaluated dysregulation of miRNAs in the MCT PAH rat model. Our results demonstrated dysregulation of miRNAs in this animal model that resembled dysregulation reported in specimens from human subjects with PAH (See Table 1). Two differences that occurred in the current study were downregulation of miR-126 and miR-145 in MCT PAH rats, which had previously been reported as upregulated in human iPAH specimens and BMPR2 deficient mice [12]. Changes in these two miRNAs in MCT PAH rats have not been previously reported. The finding of reduced expression in miR-145, which has often been considered a smooth muscle cell (SMC) specific miRNA [34, 35], in MCT PAH, was surprising since this model has been associated with a vigorous pulmonary artery SMC proliferative response. Closer examination of the data in a study by Bockmeyer and colleagues suggest that our results in MCT PAH rats may not be incongruent with human PAH specimens after all [11]. In that study, miR-145 expression levels in laser microdissected concentric lesions of lung specimens from PAH patients compared to levels in unremodeled human control PA specimens were not different. Only plexigenic lesions demonstrated an increase in miR-145 expression levels. Since the MCT PAH rat model lacks plexigenic lesions, pulmonary vascular remodeling in this model would resemble the concentric lesions of human PAH. MiR-126 expression was also decreased in laser microdissected concentric and plexigenic lesions of human PAH specimens compared to expression in unremodeled human control specimens in the Bockmeyer study, consistent with our findings in MCT PAH. Although expression of miR-204 and miR-328 was variable in lung and PA of the current study, our findings were generally congruent with published data in human PAH specimens [13, 14].

**Table 1:** MiRNA dysregulation in MCT PAH rats compared to results from human studies.

miRNA	MCT PAH rat model [Fold change (p value)]			Human [reference]	
	Lung	Microdissected PA	Plasma	Lung Specimen	Plasma
17	0.859 (0.1648)	1.76 (0.011)	1.39 (0.019)	down [15]	--
21	2.61 (0.018)	3.83 (0.0007)	1.59 (0.00001)	up [49, 50]	up [36]
126	0.327 (0.00001)	0.709 (0.04)	2.74 (0.019)	down[11]	--
145	0.609 (0.0001)	0.773 (0.02)	0.527 (0.112)	up or down[12]	--
150	0.467 (0.00004)	0.695 (0.001)	1.64 (0.015)	--	down [30]
204	1.19 (0.2381)	0.469 (0.008)	0.93 (0.438)	up or down [13]	up [36]
223	1.78 (0.043)	1.64 (0.023)	1.62 (0.005)	--	up [36]
328	0.719 (0.0056)	0.907 (0.110)	0.988 (0.477)	down [14]	--
424	0.294 (0.00001)	0.511 (0.0002)	1.32 (0.107)	down [20]	—
503	0.397 (0.00002)	0.588 (0.0004)	0.784 (0.131)	down [20]	--

Numerical values <1.0 reflect downregulation, whereas values >1.0 reflect upregulation of miRNA expression. Data are presented as fold change with p values in parenthesis. Findings in humans are referenced from the literature. --No published data available.

An unexpected result was downregulation of miR-150 in MCT PAH tissue samples, but not plasma. Rather, miR-150 was significantly increased more than 1.5- fold (Figs 1D and 2) in plasma from MCT PAH rats. This result differed from findings in plasma of human PAH subjects which exhibited reduced plasma levels of miR-150 that correlated with poor survival [30]. We note that the aforementioned study normalized their qPCR results to a C. elegans control, whereas we normalized our qPCR to an internal miR-103 control. To address this difference, we measured levels of miR-126, 145 and 150 in plasma of MCT PAH rats relative to saline control rats using each method of normalization. We found no difference in levels of expression relative to control for these miRNAs between the two methods. Thus, the incongruent plasma findings between MCT PAH and human PAH would imply the presence of a biological difference between these two species [30].

We also detected several other dysregulated miRNAs in plasma, including miR-17, 21, 126, 145, and 223 (see Table 1), which have potential implications for use as biomarkers in PAH. MiR-21 and 223 have been reported as upregulated in human blood samples from PAH subjects [36]. We do not know the much about miR-223 dysregulation in humans with PAH, but data showing upregulation of miR-223 by 1.5-fold in miRNA microarray heat maps of buffy coat blood specimens of human subjects with PAH are encouraging [36]. Preliminary results with a limited number of human PAH lung specimens in our laboratory have shown a more modest increase (~1.25 fold) in miR-223 expression (data not shown).

MiR-223 was first characterized in the hematopoietic system and has been considered a myeloid-specific miRNA [34, 35, 37, 38]. Subsequently, dysregulation of miR-223 has been demonstrated in animal models and humans with acute myocardial infarction and heart failure [31, 32]. These observations led us to evaluate the expression and function of miR-223 in MCT PAH by administering a specific inhibitor of miR-223 (A223) to MCT PAH rats. As shown in Fig 1, miR-223 was upregulated in lung, PA, and RV of MCT PAH rats. We successfully and specifically reversed the dysregulation of this miRNA in MCT PAH rats, although the reduction did not attenuate MCT PAH. Our data showed a reduction in miR-223 expression by A223 administration in PA and lung in MCT PAH to levels measured in naïve rats, whereas, A-control administration did not. One explanation may be incongruent transfection of PA and lung as compared to RV. We do not know if successful transfection of RV with A-223 is required for attenuation of MCT PAH. However, since hemodynamically and clinically, right ventricular systolic pressure is equivalent to pulmonary artery systolic pressure, it is highly unlikely that the reduction in miR-223 expression in lung and PA specimens induced by A-223 to naïve rat levels reduced PASP since no changes in RV mass or RVSP were detected (Fig 4).

Mutations in the BMPR2 gene, a member of the transforming growth factor beta (TGF β) signaling superfamily, have been identified as a cause of heritable PAH (HAPH), and also play significant roles in some forms of idiopathic PAH [21–23, 25, 39]. BMP proteins, via BMPR2, activate the canonical Smad1/5/9 signaling cascade, whereas TGFβ1 proteins activate the Smad 2/3 pathway. Mutations in BMPR2 disrupt the balance between these two pathways, leading to increased TGFβ1 and decreased BMPR2 signaling [23]. Alterations in miR-17, 21, and 145 expression levels have been associated with disrupted BMPR2 signaling [12, 15, 40]. Disrupted BMPR2 signaling is also a feature of the MCT PAH model. Prostacyclins, which play a major role in therapy of severe IPAH and HPAH, inhibit TGFβ1 signaling and enhance BMP/BMPR2 signaling [41, 42].

We reported that overexpression of hPGIS by an AAV vector delivered noninvasively to rat lung attenuated MCT PAH (Fig 5A and 5B) [33]. Lung total RNA was harvested from residual paraffin sections of MCT PAH control and MCT PAH-hPGIS rats from that study for miRNA analysis. We demonstrated that miRNAs dysregulated in MCT PAH were restored to levels measured in naïve control rats by hPGIS overexpression. Notably, levels of miRs 17, 21, and 223 were all significantly reduced and levels of miR-424 (mmu-miR-322, the mouse/rat ortholog for hsa-miR-424) and miR-503 were significantly increased (Fig 5). A223 inhibition of miR-223 did not attenuate MCT PAH, whereas hPGIS through modulation of multiple miRNAs did. Since miRNAs have been shown to act complementarily in disease [43], targeting a single miRNA may not be adequate to attenuate PAH.

We note that our comparison of miRNA levels in MCT PAH paraffin sections was subtly different from fresh MCT PAH specimens. MiR-17 was upregulated in the paraffin sections, but not in the fresh specimens, whereas miR-126, 145, 150 and 328 were not significantly downregulated in the paraffin sections as compared to the fresh specimens. These differences may reflect differences in sample size (n = 8 fresh specimens; n = 4 paraffin sections), differences in severity of PAH, or strain differences. The severity of PAH was greater in the Sprague Dawley rats from which the fresh specimens of the current study were obtained as compared to the Fischer 344 rats from which the paraffin sections were obtained (Figs 4, 5A and 5B) [44, 45]. Because A223 administration to MCT PAH rats reduced miR-223 levels to levels in naïve rats, but did not attenuate MCT PAH, we hypothesized that restoration of BMPR2 signaling was a requirement for attenuation of MCT PAH. Our data (Fig 6) was consistent with this concept since BMPR2 mRNA was restored to normal levels in lung specimens from MCT PAH rats with AAV-mediated overexpression of hPGIS, but not in MCT PAH rats that received A223.

Dysregulation of IGF1R expression is a feature of the chronic hypoxia model of PAH, in which downregulation of miR-328 correlates with upregulation of IGF1R [14]. Overexpression of miR-328 in transgenic mice suppressed IGF1R expression levels and attenuated hypoxic PH. Based on TargetScan predictions, miR-223 binds to the seed region of IGF1R at position 234–241. Overexpression of miR-223 has been shown to downregulate expression of IGFR1 in HeLa, leukemia, hepatoma, and human embryonic stem cells [46–48]. In the current study, miR-328 expression was modestly reduced in lung, but not PA, of MCT PAH rats, whereas miR-223 was consistently upregulated in all tissues and plasma. Consistent with upregulation of miR-223, levels of IGF1R mRNA were downregulated in PA of MCT PAH rats, whereas the decreased miR-328 expression would have been expected to increase IGF1R mRNA levels. No dysregulation of IGF1R mRNA levels was detected in lung specimens from MCT PAH rats with A223 administration or hPGIS expression compared to controls. We were unable to evaluate IGF1R mRNA in PA from residual paraffin embedded lung specimens with our current methodology.

In conclusion, we demonstrated that miRNA dysregulation in MCT PAH rats resembles dysregulation reported in human PAH specimens. We also report downregulation of miR-223 in MCT PAH with specific inhibition of miR-223 that failed to attenuate MCT PAH, whereas overexpression of hPGIS reversed dysregulation of multiple miRNAs in lung specimens and attenuated MCT PAH [33]. We have not explored all of the possibilities with individual miRNA inhibitors or mimetics to fully establish the link between individual dysregulated miRNAs or groups of dysregulated miRNAs to alter BMPR2 or other signaling pathways, but the use of a limited profile of miRNAs would appear to make such interventions feasible. These data suggest that targeting of specific miRNAs or groups of miRNAs may identify which signaling pathways are important for development and treatment of PAH. The stable and/or protected release of miRNAs into plasma also presents an opportunity to develop single miRNAs or panels of miRNAs as biomarkers for this very complex disease.
